# Recurrent Autoimmune Encephalitis in a Patient With Autoimmune Polyendocrine Syndrome Type 1

**DOI:** 10.1155/crcc/6086023

**Published:** 2026-05-18

**Authors:** Jason T. Stemple, Kamal Abulebda, Grace F. Ryan, Nadine G. Haddad, Alden F. Dewey, Jennah C. Foltz, Laurence E. Walsh, Riad Lutfi

**Affiliations:** ^1^ Division of Pediatric Critical Care, Department of Pediatrics, Indiana University School of Medicine, Indianapolis, Indiana, USA, indiana.edu; ^2^ Division of Pediatric Endocrinology and Diabetes, Indiana University School of Medicine/Riley Hospital for Children, Indianapolis, Indiana, USA; ^3^ Division of Medical & Molecular Genetics Riley Children′s Health, Indiana University, Indianapolis, Indiana, USA, indiana.edu; ^4^ Department of Neurology, Genetics, and Pediatrics, Indiana University School of Medicine, Indianapolis, Indiana, USA, indiana.edu

## Abstract

Autoimmune polyendocrine syndrome Type 1, also known as autoimmune polyendocrinopathy–candidiasis–ectodermal dystrophy (APS‐1/APECED), is a rare monogenic autoimmune disorder with increasing recognition of neurologic manifestations. We report a critically ill adolescent female with APS‐1 and a prior history of autoimmune encephalitis who presented with acute encephalopathy and seizures. Electroencephalography demonstrated focal seizures superimposed on ictal–interictal continuum activity, and brain MRI revealed cytotoxic lesions of the corpus callosum. High‐titer glutamic acid Decarboxylase 65 (GAD65) antibodies supported a relapse of autoimmune encephalitis after other potential etiologies were excluded. The patient demonstrated marked clinical improvement following aggressive immunotherapy, including plasma exchange, high‐dose corticosteroids, and rituximab. This case highlights the diagnostic and management challenges of autoimmune encephalitis in patients with APS‐1, and to our knowledge, represents the first reported case of recurrent autoimmune encephalitis in this population with a favorable response to early immunomodulatory therapy.

## 1. Introduction

Autoimmune polyendocrine syndrome Type 1 (APS‐1) is a rare autosomal recessive disorder caused by loss‐of‐function mutations in the autoimmune regulator (*AIRE*) gene, resulting in defective immune tolerance and multisystem autoimmunity. The classic clinical triad includes hypoparathyroidism, primary adrenal insufficiency (Addison′s disease), and chronic mucocutaneous candidiasis. Accordingly, APS‐1 is also referred to as autoimmune polyendocrinopathy–candidiasis–ectodermal dystrophy (APECED) [1].

Although endocrine and cutaneous manifestations predominate, neurologic complications, including autoimmune encephalitis, are rare but potentially life‐threatening [2]. The management of neurologic complications in patients with APS‐1 is particularly challenging due to overlapping metabolic, infectious, and autoimmune contributors to encephalopathy.

## 2. Case Presentation

A 17‐year‐old female with known APS‐1, complicated by adrenal insufficiency, hypoparathyroidism, Type 1 diabetes mellitus (T1DM), Hashimoto thyroiditis, and prior autoimmune encephalitis, presented with fever (39.0°C), sore throat, severe headache, vomiting, and progressive altered mental status over 24 h. During transport to an outside hospital emergency department (ED), she became unresponsive with preserved eye opening and experienced multiple episodes of emesis. There was no history of ingestion or prior seizures.

Initial evaluation in the ED included a negative head CT. Laboratory studies demonstrated leukocytosis, hypokalemia, and hypocalcemia (ionized calcium level was 0.9 mmol/L), and normal sodium and glucose, whereas lumbar puncture findings were reassuring. She was treated with antibiotics, calcium repletion and stress‐dose steroids. Due to ongoing vomiting and concern for airway protection during transport, the patient was intubated and transferred to a tertiary care pediatric intensive care unit (PICU).

**Figure 1:**
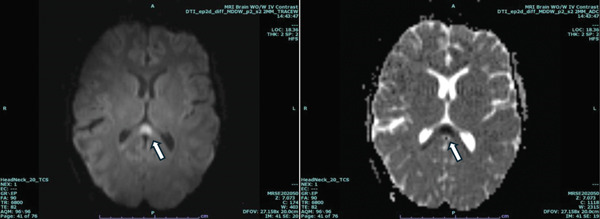
Axial MRI images of brain with arrows showing cytotoxic lesion of the corpus callosum (CLOCC).

On arrival to the PICU, the patient remained intubated on minimal sedation. She was agitated, nonpurposeful, and did not follow commands. Neurologic examination revealed episodic bilateral upper extremity hypertonicity with fist clenching and elbow flexion, followed by abrupt hypotonia. Pupils were equal and reactive, and no clonus was observed. Continuous EEG demonstrated a focal seizure arising from the right temporal lobe, ictal–interictal continuum activity, and bilateral temporal epileptiform discharges. She was loaded with levetiracetam and transitioned to maintenance therapy, after which no further electrographic seizures were observed. Brain MRI revealed a cytotoxic lesion of the corpus callosum (CLOCC) and punctate T2/FLAIR hyperintensity in the left globus pallidus internus, “findings were interpreted as nonspecific and may be associated with a wide range of conditions, including seizures, infection, metabolic disturbances, or inflammatory changes (Figure 1).” An extensive infectious workup was undertaken. A CSF meningoencephalitis (ME) panel was performed and was negative. CSF bacterial cultures were also negative. Serum and CSF studies for viral and systemic infections—including HIV, HSV, hepatitis B, hepatitis C, tuberculosis, and West Nile virus—were all negative. Respiratory viral panel testing was positive for rhinovirus/enterovirus, which was felt to be incidental and not explanatory of the neurological presentation. Additional testing showed negative Group A Streptococcus (GAS) testing and a negative urine culture. Inflammatory markers, including CRP and ESR, as well as white blood cell count, were elevated.

Autoimmune testing revealed a markedly elevated serum GAD65 antibody (> 250 IU/mL) and positive CSF GAD65 antibody 0.06 nmol/L (reference range < 0.02 nmol/L); the remainder of the autoimmune encephalitis panel was negative.

During the prior episode about 2 years prior to her current admission, the patient initially had dysphagia and limited mobility requiring wheelchair use, later developing lethargy and severe ophthalmoplegia, followed by an episode of unresponsiveness with hypercarbic respiratory failure necessitating emergent intubation. At that time, brain MRI, EEG, and infectious studies were unremarkable. CSF analysis demonstrated five unique oligoclonal bands and serum GAD65 antibody positivity at 500 IU/mL with CSF level at 0.4 nmol/L (reference range < 0.02 nmol/L). Pan‐CT imaging showed no evidence of malignancy. During a 26‐day hospitalization, she was treated with high‐dose corticosteroids, intravenous immunoglobulin, plasma exchange, and subsequent rituximab. At discharge, ophthalmoplegia had largely resolved, extremity spasticity had improved with mild residual ankle clonus, she was tolerating a regular diet, and encephalopathy had nearly resolved. Rapid genome sequencing was completed during this prior admission. Compound heterozygous AIRE variants were found, confirming the diagnosis of APS‐1/APECED. (Table 1).

**Table 1:** Results of genetic testing.

Disease	Inheritance pattern	Gene/variant	Variant type	Genotype	Inherited From	Variant classification
Autoimmune polyendocrinopathy syndrome Type I	Autosomal dominant/autosomal recessive	*AIRE*: c.967_979del, p.L323Sfs ^∗^51	Sequence variant	Heterozygous	Mother	Pathogenic
Autoimmune polyendocrinopathy syndrome Type I	Autosomal dominant/autosomal recessive	*AIRE* (HG38): chr21:44285927–44287459 DEL (1.53 kb)	Copy number variant	Heterozygous	Unknown	Pathogenic

The differential diagnosis in our patient was broad and included, but was not limited to, adrenal crisis, metabolic derangements, primary seizure disorder, and infectious etiologies. Adrenal crisis was considered unlikely, as the patient had normoglycemia, normal blood pressure, and no evidence of hyponatremia or hyperkalemia. Metabolic abnormalities—particularly hypocalcemia (ionized calcium 0.9 mmol/L)—were also considered a potential contributor to seizure activity. However, despite aggressive correction of calcium levels, there was no meaningful improvement in the patient’s mental status or seizure activity.

A primary seizure disorder was also considered. Although seizure control was achieved, the patient′s mental status remained persistently altered, with a waxing and waning examination characterized by fluctuations between hypertonicity and hypotonia. This suggested that seizures alone did not fully explain the degree of encephalopathy.

An extensive infectious workup, including cerebrospinal fluid ME panel and consultation with the infectious disease service, was unrevealing. Autoimmune encephalitis was therefore considered after exclusion of these alternative etiologies, particularly in the context of a prior similar presentation and the presence of GAD65 antibodies in both serum and CSF. Although the absolute titers in our case are lower than those reported in some series of GAD65‐associated neurological syndromes, we interpreted these findings within the broader clinical context. Specifically, the combination of clinical presentation, neuroimaging findings consistent with inflammation, positive serum and CSF GAD65 antibodies, and a similar prior episode supported autoimmune encephalitis as the leading diagnosis. This multidisciplinary assessment led to consensus among consulting teams to proceed with immunomodulatory therapy with plasma exchange (seven sessions), high‐dose IV methylprednisolone (1 g/day for 5 days), and rituximab following completion of plasma exchange. Reanalysis of the genome data from the prior admission was conducted with no change in result. This confirmed that there were no other identifiable genetic factors contributing to the recurrent autoimmune encephalitis.

Within 48 h of treatment initiation, the patient′s mental status improved dramatically, and she was extubated after two sessions of plasma exchange with continued gradual neurologic recovery. No further seizures occurred. She was discharged home on hospital Day 12 on maintenance levetiracetam, with close longitudinal follow‐up, including regular neurological assessments and multidisciplinary care involving neurology and endocrinology. We also agree that serial monitoring of GAD65 antibody titers can be helpful as an adjunct to clinical assessment, recognizing that titers may not always correlate perfectly with disease activity but can still provide useful trends over time.

Regarding relapse prevention, an active discussion supporting the role of maintenance immunotherapy in recurrent or relapsing cases was undertaken. In our patient, given the recurrence, we agree with neurology that B‐cell–depleting therapy such as rituximab administered at approximately 6‐month intervals represents a reasonable strategy to reduce relapse risk, as supported by emerging practice in autoimmune encephalitis and GAD65‐associated neurological syndromes.

## 3. Discussion

This case illustrates the complexity of diagnosing acute encephalopathy in patients with APS‐1 in the intensive care unit. Metabolic derangements, adrenal crisis, infection, and autoimmune encephalitis may coexist or mimic one another, complicating diagnostic evaluation. Although GAD65 antibodies are frequently detected in APS‐1, clinically significant GAD65‐associated autoimmune encephalitis remains rare and the causal relationship between GAD65 antibodies and encephalitis remains uncertain [3, 4]. In this patient, the presence of focal seizures, ictal–interictal EEG patterns, elevated GAD65 antibody titers, and a favorable response to immunotherapy support a relapse of autoimmune encephalitis.

**Figure 2:**
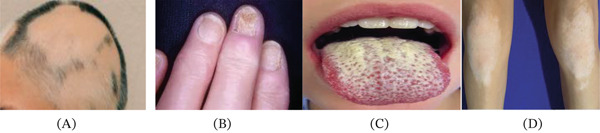
Clinical images depicting mucocutaneous and dermatologic manifestations in (A) alopecia areata, (B) nail dystrophy, (C) *Candida* thrush affecting the tongue, and (D) vitiligo.

APS1 is a rare monogenic autoimmune disorder caused by loss‐of‐function mutations in the *AIRE* gene. *AIRE* deficiency impairs central immune tolerance in the thymus, resulting in the peripheral escape of autoreactive T lymphocytes and the generation of multiple cytokine‐ and tissue‐specific autoantibodies [1, 4]. The classic clinical triad of APS‐1 includes chronic mucocutaneous candidiasis, hypoparathyroidism, and primary adrenal insufficiency (Addison′s disease). Additional endocrine manifestations frequently include T1DM and autoimmune thyroid disease, whereas nonendocrine features may include alopecia, vitiligo, dental enamel hypoplasia, and other ectodermal abnormalities. These manifestations often begin in childhood; however, T1DM most commonly presents in the third to fourth decade of life rather than in early childhood [5–6] (Figure 2). APS‐1 patients may also develop a wide range of nonendocrine autoimmune complications, underscoring the importance of early recognition by pediatric providers to enable timely screening, prevention, and targeted therapeutic interventions.

Neurologic involvement in APS‐1 has been described primarily in case reports and small series, with a broad spectrum of manifestations [7, 8, 9]. Cerebellar ataxia associated with anti‐GAD antibodies is frequently reported. Facchini et al. described a child with combined subacute degeneration presenting with severe sensory ataxia, unusual skin hyperpigmentation, and megaloblastic anemia [10]. Demyelinating lesions and multiple sclerosis–like presentations have also been reported. In one cohort study of 44 APS‐1 patients, 10 (23%) were diagnosed with a neurologic disorder. Serum antineuronal antibodies were detected in 42% of tested patients, with GAD65 antibodies being the most common. Low titers of antibodies against glycine receptors and aquaporin‐4 were also identified; however, none of the patients in this cohort were diagnosed with GAD65‐associated autoimmune encephalitis [2]. A recent case report described anti‐GAD65 autoimmune encephalitis in a child with APS type II [11] and another recent study specifically examined the relationship between GAD65‐related temporal lobe epilepsy (TLE). The study suggests that GAD65‐TLE may share a common pathophysiology and should be included among APS‐III diagnostic criteria [12].

Anti‐GAD antibodies have been implicated in a range of neurologic syndromes, including stiff person syndrome, cerebellar ataxia, refractory epilepsy, and limbic and extralimbic encephalitis [13]. Although anti‐GAD65–associated encephalitis has been reported in adult populations, pediatric cases remain rare. Glutamic acid decarboxylase is essential for the synthesis of the inhibitory neurotransmitter *γ*‐aminobutyric acid (GABA); thus, antibodies targeting GAD may reduce GABA availability, shifting the balance toward excitatory neurotransmission and increasing seizure susceptibility [14].

To our knowledge, this represents the first reported case of recurrent GAD65 antibody–associated autoimmune encephalitis in a patient with APS‐1. The co‐occurrence of these autoimmune conditions suggests shared genetic, immunologic, and/or environmental mechanisms underlying organ‐specific autoimmunity. Early recognition and prompt initiation of immunomodulatory therapy are critical to preventing prolonged neurologic injury and improving outcomes in critically ill patients with autoimmune encephalitis.

## Funding

No funding was received for this manuscript.

## Consent

All the patients allowed personal data processing, and informed consent was obtained from all individual participants included in the study.

## Conflicts of Interest

The authors declare no conflicts of interest.

## Data Availability

The data that support the findings of this study are available on request from the corresponding author. The data are not publicly available due to privacy or ethical restrictions.
